# Strengthening Governance for Universalising Primary Oral Health Care: Perspectives from Karnataka, India

**DOI:** 10.12688/wellcomeopenres.24152.1

**Published:** 2025-06-02

**Authors:** Rajeev Rudrappa Basapathy, Manu Raj Mathur

**Affiliations:** 1 Institute of Public Health Bengaluru, Bengaluru, Karnataka, India; 2 Public Health Foundation of India, New Delhi, Gurugram, Uttar Pradesh, 122002, India

**Keywords:** Oral healthcare delivery system, Karnataka, Governance, Oral Health, India

## Abstract

**Background:**

Governance is central to health systems, and achieving Universal Health Coverage (UHC) relies on strong, sustainable systems. However, despite UHC's broad health goals, issues like oral health receive inadequate attention, signalling inequity in health systems. In India, oral diseases are rising, yet oral health remains a low political priority, reflecting weak governance and limited state commitment to health equity. This study analysed governance factors within Karnataka's public oral healthcare system through the lens of Siddiqi’s health governance assessment framework.

**Methods:**

In this exploratory qualitative study, in-depth interviews with twenty stakeholders, including administrators and program implementers, explored enablers and constraints at policy and operational levels. Data analysis was guided by the governance framework developed by Siddiqi and colleagues.

**Results:**

The findings indicate that challenges persist while Karnataka’s oral health governance benefits from a separate directorate ensuring administrative functionality. These include a lack of strategic vision for oral health, inadequate policy formulation, limited social participation, insufficient budget, workforce shortages, outdated guidelines, and inequitable oral health programs. Power dynamics, particularly with district health officers, further hinder effective governance. The study reveals a siloed approach to oral health with minimal integration into broader health programs. From planning to implementation, weak governance links reflect low political will.

**Conclusion:**

Although recent attention has been given to oral health in Karnataka, substantial reforms are necessary. These include appointing oral health personnel at primary health centres, increasing budgets, revising the Indian Public Health Standards to prioritise oral health in primary care, empowering the oral health directorate, and establishing accountability and surveillance systems. Strengthening governance in these areas is essential to advance oral health equity and contribute to UHC goals in Karnataka.

## Plain Language Summary

Oral health is an important part of overall health, but it is often overlooked in India’s public health system. In Karnataka, a southern state of India, the government has made some efforts to improve oral health services, but challenges remain in areas like funding, staffing, and reaching communities with preventive care. This study explored how oral health services are planned, delivered, and managed in Karnataka by speaking with people working in the public health system. We used a governance lens, focusing on how policies are made, implemented, and monitored.

The study found that oral health is not yet treated as a priority in health planning. Services are mostly dentist-centred and focused on treatment, with little attention to prevention. The lack of clear guidelines, data systems, and accountability makes it hard to track progress. The study recommends a shift toward community-based care, more resources, stronger leadership, and better coordination with the private sector to ensure everyone has access to quality oral health services.

## Introduction

Oral diseases, which are primarily chronic and non-communicable, affect a large portion of the global population and have significant implications for overall health. They also impose a substantial socio-economic burden, particularly in low- and middle-income countries ^1^. Despite this, oral health remains a low political priority in India and worldwide ^2– 4^. This lack of prioritisation has resulted in limited opportunities to advocate for the inclusion of oral health in UHC benefit packages. Recent global developments have provided new momentum for advancing oral health on the public health agenda. The adoption of a resolution on oral health at the 74th World Health Assembly (WHA) urged member states to integrate oral health into national policies within the UHC framework ^1^. Additionally, the International Dental Federation's Vision 2030 document proposes strategies for achieving optimal oral health in alignment with the Sustainable Development Goals (SDGs) and UHC ^5^. In May 2022, the 75th WHA endorsed a global strategy on oral health, aiming to make high-quality oral healthcare services affordable, available, and accessible to all ^6, 7^. These developments collectively call for stronger governance, particularly in policy-making and program design, to achieve Universal Oral Health Coverage (UOHC).

The World Health Organisation (WHO) has identified six key building blocks of health systems: Governance, Financing, Human Resources, Information, Medicines and Technologies, and Service Delivery ^8^. Governance, in particular, plays a central role by influencing all other building blocks ^9^. Various frameworks have been developed to assess health governance, drawing from disciplines such as New Institutional Economics and International Development, and theories like Social Accountability and Institutional Analysis ^10^. These frameworks offer different perspectives on how to strengthen governance in health systems, yet the debate over the most effective approaches, particularly in the context of integrating specific health issues like oral health into broader health policies, remains largely unexplored.

Karnataka is a state in Southern India, recognised for its relatively robust health system. The demographic and geographic diversity poses unique challenges in addressing health disparities, particularly in rural and underserved areas where access to quality healthcare remains uneven. As a result, the scale and complexity of public health issues in Karnataka demand targeted interventions to bridge gaps in healthcare delivery and ensure equitable access to services across its vast expanse ^11, 12^. However, oral health has received limited attention, as evidenced by the lack of focus on oral health in recent reports on the state’s progress toward the SDGs ^12^. Effective leadership and strong governance are essential for achieving health equity, yet the lack of commitment to oral health in Karnataka is particularly concerning given the state’s rising burden of oral health issues. These conditions disproportionately affect marginalised communities, who already face barriers to accessing care. The WHA resolution emphasises the importance of developing tailored oral health policies and programs that meet the need for comprehensive preventive, promotive, rehabilitative, and curative services. This aligns with WHO's Global Oral Health Strategy 2030, which prioritises universal health coverage through equitable and integrated health systems, including oral health as a critical component ^7^. Similarly, India's National Health Policy of 2017 stressed the significance of preventive and promotive healthcare, advocating for a shift from curative services to a more proactive approach in addressing the broader determinants of health ^13^. Together, these frameworks highlight a global and national commitment to strengthening oral health systems to achieve better health outcomes for all. However, there has been limited analysis of how oral health is integrated into national and state health policies in India, raising questions about Karnataka’s efforts to align with this global mandate. While some reports have touched on the subject ^14^, a comprehensive analysis is lacking. Recognising this gap, this study analysed the governance factors influencing oral healthcare in Karnataka. Specifically, it sought to explore stakeholders’ perceptions of oral healthcare governance and examine how governance impacts the integration of oral health services into UHC packages.

## Methods

This study was guided by the Health Governance Assessment Framework developed by Siddiqi and colleagues, which focuses on Low- and Middle-Income Countries (LMICs) ^15^. This analytical framework tool allows assessment at national (policy formulation) and sub-national levels (policy implementation) and also helps inform interventions that can improve health system performance. The semi-structured interview questionnaire was developed based on the frameworks of Siddiqi *et al.* and Batchelor, incorporating ten governance principles and tailoring the questions to address specific domains such as context, processes, and outcomes, ensuring relevance to Karnataka’s oral health governance landscape ^16^ ( Table 1). This inquiry focused on the governance and policy responses of state-level administrators and other stakeholders involved in implementing oral healthcare services at the district, sub-district, and village levels.

**Table 1.** Governance principles.

Sl No	Principles	Questions arising at a sub-national level
1	Strategic vision	Is there a clear, well-defined policy and long-term vision for improving health?
2	Participation and consensus orientation	How are decisions finalised, who was involved, and how were the differences reconciled?
3	Rule of law	Are laws governing the determinants of health and service provision in place and how are they enacted?
4	Transparency	Are data on finance and administration readily available and is the process transparent?
5	Responsiveness	Is needs assessment part of the resource allocation process and how are any findings used?
6	Equity and inclusiveness	Are social protection schemes in place and what processes exist to address shortcomings?
7	Effectiveness and efficiency	What are the qualities of staff and what processes exist to ensure evidence-informed policies?
8	Accountability	What is the role of the legislative body and how are its/their actions overseen?
9	Intelligence and information	What data are available to inform decision-making processes and how are they used?
10	Ethics	What mechanisms exist to promote and enforce standards in care and research?

**Figure 1.**
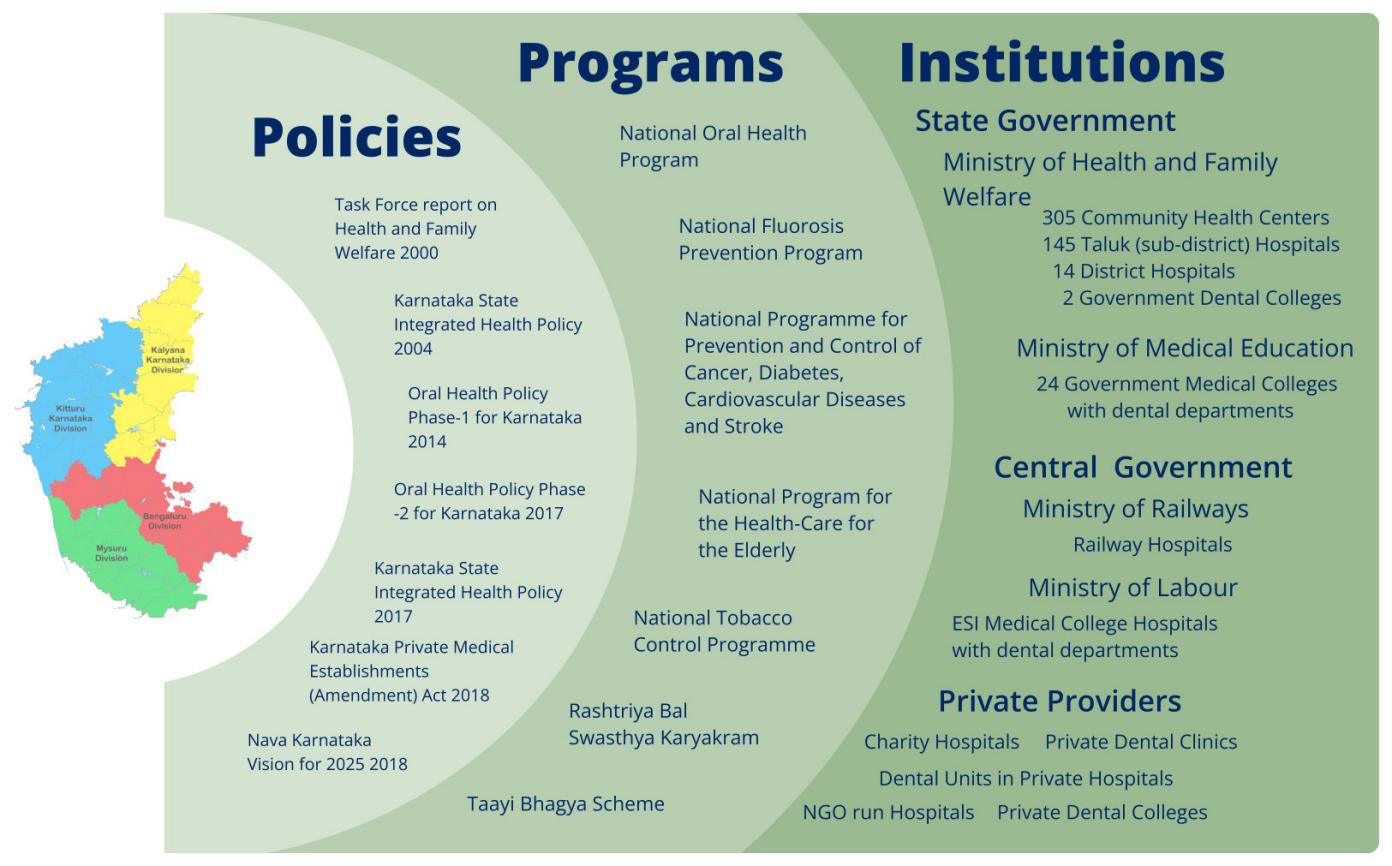
Overview of the Oral Health Ecosystem in Karnataka. This figure presents the key components shaping oral health service delivery in Karnataka. It categorises the ecosystem into three domains: Policies, Programs and Institutions.

An exploratory qualitative approach was employed due to the limited pre-existing knowledge of actors’ perspectives regarding oral health policy and program implementation ^17^. A situational analysis of Karnataka’s oral healthcare delivery system was conducted using multiple data sources, including interviews with key administrators and reviewing relevant policy and program documents accessible via the Karnataka state health ministry’s website. Participants were selected using a snowball sampling method, drawing from administrators, public and private sector dentists, academicians, dentists in medical colleges, and program implementers ( Table 2). To ensure a comprehensive perspective, participants were purposively selected from the four administrative divisions of Karnataka ( Figure 1). Data was collected through in-depth telephone and in-person interviews, which lasted between thirty and ninety minutes.

**Table 2.** Participants’ details.

Sl No	Participants (Identity code)		Sample	
1	Administrators		5	
2	Sl No	Dentists working in the public sector		9
	1	District Hospital	4	
	2	Taluk Hospital	3	
	3	Community Health Centre	2	
3	Private dental practitioner		1	
4	Urban PHC dentist		1	
5	Academician working at a private dental college		1	
6	Dentists working in Medical College Hospital		2	
7	Program implementer		1	
8	Total		20	

Data collection took place between October and December 2021. Data collection continued until saturation was reached, as determined by both authors. Most interviews were audio-recorded, with handwritten notes taken for participants who did not consent to recording. The recordings were transcribed and anonymised, with unique codes assigned to mask participant identities. Data analysis was conducted using the framework method with an Excel spreadsheet to organise responses by the framework’s domains ^18^. Themes were identified using both inductive and deductive coding methods. Both authors reviewed the emerging matrix to identify patterns in responses and potential biases.

## Results

### Oral healthcare service delivery in Karnataka

In Karnataka, a comprehensive network of oral health policies, programs, and institutions works in tandem to address the state's oral health challenges. Policies set the strategic framework for public health goals, focusing on accessibility, prevention, and equitable healthcare delivery. Programs translate these policies into actionable initiatives. Institutions implement these programs and provide the necessary infrastructure and logistical support. Together, these components form a robust system aimed at improving oral health outcomes across the state ( Figure 1).

### Policies

Seven policies were identified which were relevant to oral health from the year 2000 to 2021. Karnataka’s oral healthcare framework is guided by several key policies and reports. The Task Force Report on Health and Family Welfare emphasised strengthening the public health system, including oral healthcare. The Karnataka State Integrated Health Policy promoted universal healthcare, integrating oral health within its framework. The Oral Health Policy Phase-1 and Phase-2 focused on expanding dental care access, from preventive measures to specialised treatments. The Karnataka Private Medical Establishments (Amendment) Act targeted accountability and standardisation in private healthcare services. Lastly, the Nava Karnataka Vision 2025 envisioned a healthier state by enhancing healthcare quality, including oral health, for all citizens.

***Institutions*.** Oral health services in the public sector were delivered through the three-tier primary healthcare system through the 305 Community Health Centres (CHC), 145 Taluk (sub-district) Hospitals (TH), 14 District Hospitals (DH), 2 government dental colleges, 24 government medical colleges which have dental clinics and 1 Employee State Insurance (ESI) dental college and 1 ESI medical college hospital. Clinical oral health services were unavailable at the Sub Centres (SC) and Primary Health Centres (PHC), which are the most accessible and first point of contact health centres. Oral healthcare delivery was predominantly limited to curative outpatient services. Besides this, a range of private players delivered care.

### Programmes

The National Oral Health Programme (NOHP) was initiated through the National Health Mission (NHM) in 2014, and subsequently, the programme was started in Karnataka state in 2016 with a separate directorate. A recognised post of Deputy Director (DD) was created to head the state’s NOHP in 2017, but a gynaecologist was appointed for the DD’s post, which was replaced by a dentist in 2021. At the district level, the programme was implemented through the District Health Officers (DHS), who were medical professionals. Divisional Nodal Officers (DNO) posts were created in the four divisions of Karnataka to assist DD. The DNO posts were additional charges given to the senior dental officers in each administrative region. At the district level, the District NOHP Officer post was an additional role given to the existing dentist working at the DH, who oversaw the delivery of oral health services from the DH, TH, and the CHCs. Besides this, there was a state NOHP consultant. 425 dentists were working in the public sector at DH, TH, and CHCs, with 35 of them having additional administration roles. Health and Wellness Centres replaced SC as part of the Ayushman Bharat program, where oral health was identified among the focus twelve health issues ^19^. These centres were managed by health workers under the supervision of the medical officer at the PHC. Oral health was also addressed through other programmes such as the National Fluorosis Prevention Programme, National Programme for Prevention and Control of Cancer, Diabetes, Cardiovascular Diseases and Stroke (NPCDCS), National Programme for Health Care for the Elderly, National Tobacco Control Programme, Rashtriya Bal Swasthya Karyakram *(*RBSK *)* and Taayi Bhagya Scheme ^20^, a maternal programme where pregnant women are screened by health workers for oral diseases in their second trimester and referred to the nearest dentists at DH, TH or CHC. Karnataka state launched a standalone oral health programme called Danta Bhagya Yojane in 2014–15. The programme provided dentures to people above 60 from economically backward sections ^21^. The programme was implemented as a public-private partnership with 43 private dental colleges, since many DHs did not have the necessary infrastructure to provide these services.

### Governance factors

***Strategic vision*.** One of the administrators aptly summarised the current state of oral health governance: *"Right now, we have a little bit, but not all"* (Senior Administrator). This reflects the fragmented approach to oral health in the public sector, where it is managed as a vertical program with little convergence with other health initiatives. The lack of a strategic vision to prioritise oral health is evident as more attention is given to other health programs.

*Other programs are there... Malaria officers and family planning officers are there. Why not oral health? Oral health plays a major role; it should be prioritised. Otherwise, it will remain just on paper* (Dentist at the District Hospital 1). This statement highlights the absence of leadership in integrating oral health into broader health strategies.

Respondents viewed the oral health policy as a critical mechanism for streamlining resources, increasing budget allocations, and addressing workforce shortages. However, the lack of oral health services at PHCs is seen as a failure in strategic planning. Furthermore, the policy formulation process was perceived as overly centralised, with little involvement of key stakeholders such as regional administrators. This top-down approach reflects a missed opportunity for building a shared strategic vision that could ensure the integration and prioritisation of oral health within the public health system. 

Strengthening governance with a clear, inclusive strategic vision is essential to effectively address oral health challenges, moving beyond sporadic efforts to a coordinated, long-term approach.

***Participation and consensus orientation*.** Participants emphasised that community participation in oral health is largely shaped by an awareness of available services and the infrastructure at healthcare facilities. Social participation was closely tied to key health system values such as trust, ethical care, patient feedback mechanisms, and misconceptions about the quality of care in public facilities.

*People are not aware that dentists are available in the centers. For them to use the services, we need to inform them that dentists are here, right?* (Junior Administrator 2). This reflects the importance of communication and transparency in fostering trust and increasing community engagement. Without clear messaging, people often mistrust the system, assuming it is dysfunctional or inadequate.

*People will think that nothing here is working. That’s one reason they are not coming. The first notion is that better care is always in private* (Junior Administrator 1). This highlights the widespread perception that private healthcare offers superior services, further diminishing participation in public oral health programs.

The need for a consensus-oriented approach, where the public is involved in shaping health services, emerged as a crucial governance factor. Enhancing participation by addressing these trust and communication gaps, alongside ensuring service quality, is essential for building a more inclusive and responsive oral health system. A lack of community input not only affects service uptake but also weakens the potential for collaborative solutions to improve public health care delivery.

***Rule of law*.** The governance of oral health in Karnataka is shaped by different legal frameworks across institutions. For example, the ESI hospital’s dental branch is governed by the Dental Council of India, while the dental department in medical colleges falls under the purview of the National Medical Council. In medical colleges, dentistry is often viewed as just one of many departments, and the lack of recognition of dental specialities impacts recruitment patterns, with certain specialised dentists unavailable, limiting the range of services offered.

The Indian Public Health Standards (IPHS) serve as the key guiding document for primary healthcare, dictating workforce and infrastructure decisions. However, the IPHS has also been used as a legal justification for limiting the inclusion of dental services at the primary care level. For example, workforce requests to improve dental services have been dismissed based on IPHS guidelines:

*The state government looks at IPHS guidelines. It (IPHS) doesn’t state that a dentist post is mandatory at the PHC level. Since IPHS has not prescribed that a dental surgeon is required, we do not have one* (Junior Administrator 3).

This demonstrates how the strict adherence to existing legal frameworks without updating them to reflect evolving healthcare needs can hinder the integration of oral health services. The rule of law, while essential for standardisation, can become a barrier to progress if not aligned with current health priorities. Legal and policy reforms are necessary to ensure that oral health is adequately represented within public health systems, especially at the primary care level.

***Transparency*.** Administrators voiced concerns about poor monitoring and evaluation of clinical services, although there were signs of improvement with the establishment of the NOHP and designated posts like DD and DNOs to oversee monitoring activities. Financial transparency improved under NOHP, as it streamlined the budgeting process, reducing delays in the release of funds. Annual funding, which began in 2017–18 with a budget of Rs 2.5 Crores (0.3 million USD), steadily increased to Rs 5.3 Crores (0.64 million USD) by 2020–21. This was seen as a positive step, particularly as delays in funding releases had been a persistent issue. NOHP supports the state government in sharing the salaries of dentists and dental auxiliaries, procuring equipment, and supporting outreach and awareness activities.

However, despite the improvements, transparency challenges remain, particularly with fund utilisation. Fixed budgets for the maintenance of DH, TH, and community health centres (CHC) were often underutilised, leading to critical remarks during review meetings and potentially affecting subsequent funding.

An administrator explained the issue: *We send the budget requirement by April every year, but the funds are released in the following February, ten months later. We are expected to use all the funds within two months before preparing the next annual budget. So, a lot of money remains unused* (Junior Administrator 1).

This delay in fund release and the unrealistic timeline for expenditure hampers effective financial management, highlighting the need for more transparent and efficient financial planning processes to ensure timely fund allocation and utilisation, a crucial aspect of good governance.

***Responsiveness*.** The implementation of the NOHP is carried out at the district level, with the DHO responsible for allocating funds across various health programs. However, oral health often competes with other health issues for budgetary attention, and power dynamics between medical and dental personnel further influence these financial decisions.

While some DHOs and District Surgeons (DS) recognised the importance of oral health, others did not, leading to inconsistent support. The lack of formal recognition of key administrative roles, such as the DNO, has created tensions and revealed power imbalances between medical and dental administrators, directly impacting oral healthcare delivery.

*The DNO is not a permanent post. It is an additional charge given to personnel in existing posts. The DNO operates under the DS, and if a DNO wants to conduct an inspection, the DS may object, claiming it would disrupt hospital work. The dental department lacks the authority and autonomy to act independently. We are under the DS’s control and have to seek approval from higher authorities. Our hands are tied, yet we are expected to swim. This leads to failures* (Junior Administrator 2).

Responsiveness in governance is further tested during needs assessments and gap analyses, which focus on infrastructure in public facilities and are conducted annually. These assessments are carried out at the district level by the NOHP District Program Officer, reviewed by the DNO, and assessed against the IPHS guidelines. The combined state-level assessment is finalised at the annual Program Implementation Plan (PIP) meeting, held around November–December, before being sent to the NHM for approval. This process demonstrates an effort to respond to infrastructure needs; however, the hierarchical and bureaucratic constraints slow the response to evolving needs in oral healthcare, pointing to the need for more autonomous decision-making and authority at the district level for improved responsiveness.

***Equity and inclusiveness*.** The absence of social insurance for oral health services has led to increased out-of-pocket expenditures, as most dental treatments are not covered by state-run insurance programs. A common perception that dentistry is primarily cosmetic further hinders efforts to provide equitable oral health services. The Ayushman Bharat Arogya Raksha Kavach (ABARK), a central government health assurance program, offers health benefits packages but only covers tertiary-level, life-saving procedures such as oral cancer treatment and cleft surgeries. Although Karnataka was one of the first states to implement ABARK, it remains limited in scope for oral health, with discussions underway to include more dental procedures in the program.

The lack of primary oral healthcare services at PHCs, particularly in rural areas, forces patients to seek care at secondary or tertiary facilities. This not only places a travel and financial burden on patients but also strains the capacity of higher-level healthcare centres. The overemphasis on curative treatments increases out-of-pocket expenses and adds pressure to an already stretched health system.

Oral health challenges are also region-specific. Northern Karnataka, for example, faces endemic issues like dental fluorosis and widespread betel nut chewing habit predisposing to Oral Submucous Fibrosis, both of which have serious oral health implications. While rural areas receive some attention in oral health initiatives, urban areas, especially within the public sector, are often overlooked, exacerbating inequalities in service delivery. This geographic disparity highlights the need for a more comprehensive and equitable approach to oral healthcare across different regions of Karnataka.

***Effectiveness*.** Two dentists are typically posted at DH, with 1–2 at TH, and one at CHC. However, respondents felt this staffing was inadequate for providing comprehensive oral health services. One respondent highlighted the critical need for more staff to ensure preventive services are prioritized:

*With such limited staff, we cannot focus on preventive services. We need at least 7–8 dentists at DH, 3–4 at TH, and at least 2 at CHC. Public health dentists are urgently needed for exclusive preventive work. It’s not feasible to provide regular preventive care with such minimal staff* (Senior Administrator)

The shortage of dental assistants was a universal concern among respondents. Dental assistants play a crucial role in improving the efficiency of care, but a lack of understanding among decision-makers regarding the distinct roles and competencies of different types of dental auxiliaries exacerbates the issue. A dentist from a CHC shared their frustration:

*Policy-makers don’t understand the difference between a dental technician and a lab technician, or other types of technicians. They use these terms synonymously, which creates confusion. They say, 'Lab technician is there, no? A technician is a technician, so why not use their help?* (Dentist at the Community Health Centre 2).

In terms of building capacity, the training of health workers, like ASHA workers, to recognise and refer patients with oral health issues has primarily been linked to mother and child health services. Some respondents expressed concerns about the lack of ongoing dental education. While dental staff trained medical officers in basic oral care for the NPCDCS program, there was no structured training for dentists to continuously update their skills. This gap in capacity-building measures limits the effectiveness and quality of oral health services at all levels of care.

***Intelligence and information*.** The absence of a comprehensive survey and a reliable surveillance mechanism to assess the exact burden of oral diseases was identified as a significant barrier to designing an effective and well-planned oral health program. Respondents stressed that without precise data, it is challenging to justify funding or implement targeted interventions. One participant highlighted the urgent need for both a population-based survey and a robust monitoring system to collect data from various sources:

*We need an oral health survey to determine the disease burden. It is the biggest need. It will strengthen the argument for more funding. Additionally, we require a software management system that can monitor and collect data from dental colleges, medical institutions, and private dental practitioners* (Senior Administrator).

The lack of reliable data impedes efforts to advocate for increased resources and hinders the ability to develop policies that reflect the true extent of the oral health challenges faced by the population. An integrated data collection system would allow for more accurate program planning, enabling a responsive and evidence-based approach to addressing oral health needs.

***Ethics*.** Ethical concerns have emerged in implementing programs like the RBSK, particularly regarding the oversight of parental consent when treating children for dental issues. Participants highlighted that conducting dental procedures, especially surgical interventions, without the explicit consent of parents raises serious ethical questions:

*In 2016, they instructed us to conduct camps where dentists from CHCs should go to schools and provide treatment. But how can we perform treatments, especially surgical ones, without the parent’s consent? It is unethical* (Junior Administrator 2).

Additionally, the participation of schools in these programs introduced a bioethical dimension. Schools often prioritised non-invasive procedures, like providing spectacles, over dental treatments that involved clinical intervention, reflecting hesitancy towards dental care:

*The principal would welcome the dentist but opt for spectacles over dental treatment. They would say, ‘No, no, we have a function today, we cannot do it today,’ showing clear reluctance* (Program Implementer).

This highlights the need for clearer ethical guidelines and accountability mechanisms in oral health programs to ensure that patient rights, particularly those of minors, are upheld and that schools and healthcare providers align on ethical treatment practices.

## Discussion

Governance is a critical element in identifying practical solutions to strengthen health systems, particularly in oral health. Effective governance requires not only financial resources but also administrative reforms to create a coherent strategy. This study indicates that the current governance of oral health in Karnataka is characterised by a siloed approach that often neglects the integration of dental care within the broader health system. This disjointedness stems from a lack of strategic vision for oral health, as evidenced by the existence of seven policies related to oral health in Karnataka, many of which administrators were unaware of. Furthermore, some policy recommendations appear incongruent with on-the-ground realities, lacking a rigorous connection to necessary financial and human resources for effective implementation. The presence of multiple policies, coupled with insufficient implementation guidelines, hinders progress.

The role of community involvement in health participation has seen significant advancements, particularly through the NHM’s Community Action for Health strategy. Initiatives such as the Community Planning and Monitoring of Health Systems in Karnataka have fostered the establishment of patient welfare committees at the village level, enhancing accountability and facilitating community participation ^22, 23^. However, similar to findings from South Africa's school oral health program, there is a notable absence of mechanisms that promote social participation in the oral health initiatives ^17^. This gap reflects a loose planning framework and a lack of consensus on effectively addressing the oral health burden. The governance framework must address the principle of the rule of law, particularly in revising the IPHS that designates dentistry as a desirable service at the PHC level ^24, 25^. The revision of dentistry to an essential service at the PHC level is crucial to reducing oral health inequalities since the PHC is the first point of contact for rural and underserved areas. The NHM and the NOHP have made strides in emphasising oral health by imposing obligations on state governments to adhere to central guidelines, such as the IPHS. Moreover, the NHM serves as a lever for increasing budgets, consumables, infrastructure, and workforce allocations, making it imperative that IPHS revisions are prioritised to drive significant change. In terms of transparency, the current monitoring and evaluation processes primarily focus on existing infrastructure rather than on the efficacy of service delivery. Participants emphasised the need for regular assessments of oral health profiles and service delivery data to inform planning processes. Sheik and Abimbola have highlighted the importance of viewing strong health systems as learning systems that are better at managing their resources by recognising and utilising innovations arising from community experiences ^26^. Therefore, an effective oral health intelligence system should strengthen the domestic capacity that is essential for collecting sociological, epidemiological, behavioural, and demographic data, which can aid in crafting context-specific indicators to monitor program success. Financial transparency also emerged as a concern, as delays in budget disbursement hinder effective implementation. The problem lies not in the utilisation of funds for the oral health program but in governing the inflow and spending; underutilisation of the budget can be mitigated by transitioning to outcome-oriented budgets instead of relying solely on line-item budgets ^27^.

A concerning trend observed in this study is the lack of prioritisation of oral health by certain district officials, directly affecting budget allocations. This disconnect illustrates a failure to build relationships with oral health stakeholders at the local level ^17^. The perception of oral health treatment as simplistic among medical officers further diminishes its priority within the health system. When DS and DHOs evaluate oral health care based solely on the number of cases treated, they neglect to consider the time investment required for dental procedures, which range from 15 to 45 minutes. This evaluation metric reflects a broader misunderstanding of oral healthcare delivery.

In a healthcare landscape dominated by private providers, the reliance of underserved populations on public health systems highlights equity concerns. The lack of comprehensive dental treatments within ABARK indicates a need for better understanding among decision-makers regarding the systemic health implications of oral diseases. Furthermore, the piecemeal nature of oral health services influences public utilisation of public healthcare services. Addressing the absence of oral healthcare at PHCs is essential for equity and necessitates a paradigm shift from a curative focus to a preventive one.

To enhance the effectiveness of oral health governance, strong political commitment and resource mobilisation are essential. Increasing the diversity of the oral health workforce, such as dental assistants, technicians, and speciality dentists, and augmenting the oral health budget should be considered priority actions. The establishment of a separate directorate for oral health, led by a dentist with adequate financial powers, assisted by a permanent post of DNOs, could significantly improve accountability within the sector.

Moreover, ethical treatment practices must be incorporated into planning, emphasising the bioethical perspective crucial for equitable health delivery. As noted by Chalkidou and colleagues, achieving UHC necessitates more than just prioritisation; it demands political commitment and adequate financial resources ^28^. Governance and organisational changes at the ministerial level can further support transparency and accountability, as argued by Berman and colleagues ^29^.

COVID-19 has laid bare the vulnerabilities of India's health system, particularly the challenges facing oral healthcare, which is often relegated to a low priority. Fundamental reforms such as investing in the processes of continuous consultation, coordination, and collaboration across central, state, and local levels of government, guided by an underlying adaptive governance logic, are needed ^30^. An ideal oral healthcare system should not only be equipped to offer routine care but also respond to urgent needs during emergencies ^31^. Explicit, transparent and accountable priority-setting processes that pay attention to alternative trade-offs are feasible to achieve UOHC in a resource-constrained health system ^28^. Such reforms should include a decentralised oral health data system, improving purchasing and regulation of the private sector and intersectoral delivery of health services ^31^. For a progressive realisation of UOHC, governments should develop and monitor essential indicators in the following domains: population coverage, service provision, access to care and financing ^32^.

This study has several strengths, including its application of a robust governance framework, which incorporates ten governance principles and tailors the analysis to the oral health governance landscape in Karnataka. By focusing on context, processes, and outcomes, the study provides deep, context-specific insights into governance gaps and actionable recommendations, such as revising Indian Public Health Standards, enhancing surveillance systems, and creating a separate oral health directorate. The use of semi-structured interviews adds depth to the findings by capturing diverse stakeholder perspectives, while the emphasis on equity aligns with universal oral health coverage goals. However, the study also has limitations. It primarily focuses on the public sector, overlooking the critical role of the private sector, which dominates oral healthcare in India. Further research is necessary to identify strategies for better integration of public and private services to achieve UOHC. Additionally, the study could not capture accountability mechanisms, monitoring, and evaluation of dentist performance, workforce shortages, or patient treatment outcomes, which are vital areas for future investigation. The absence of quantitative data, broader political-economic analysis, and longitudinal perspectives further limits the scope of the findings. Addressing these gaps in future research will be essential to develop a more comprehensive understanding of oral health governance in Karnataka and beyond.

## Conclusion

While oral health in Karnataka has made some progress, critical gaps remain in governance, particularly in budget allocation, workforce development, and accountability. Strengthening governance requires not just financial investment but also strategic administrative reforms. Governance reforms must prioritise regular epidemiological surveys and a robust surveillance system tailored to India’s context. Developing clinical oral health data management tools that integrate public and private healthcare providers will enhance transparency and accountability in decision-making. Standardising services across all levels of care is crucial, with the establishment of preventive services at PHCs and multidisciplinary teams of specialist dentists at tertiary levels, supported by auxiliary staff to improve service efficiency.

At the policy level, revising the IPHS to reflect oral health priorities, increasing the oral health budget, and incorporating dental medications into government pharmacies are necessary steps. Establishing a separate oral health directorate with sufficient administrative and financial powers will enhance accountability and ensure the system can meet both routine and emergency care needs. Embedding governance principles such as accountability, transparency, and community participation in oral health policies will drive Karnataka’s progress toward UOHC and strengthen the system’s capacity to deliver equitable, high-quality care.

## Ethics and consent statement

Ethics approval for this study was obtained from the SOCHARA Institutional and Scientific Ethics Committee, Bengaluru (SISEC 2021/10/1, dated 1st October 2021). Informed consent was obtained from all participants. Of the total interviews conducted, two were in-person and the remaining were conducted over the telephone. For the two in-person interviews, written informed consent was obtained after explaining the study objectives, procedures, risks, and confidentiality measures. For the telephone interviews, verbal informed consent was obtained due to the constraints imposed by the COVID-19 pandemic, which made it logistically difficult and potentially unsafe to collect written consent. The use of verbal consent was reviewed and approved by the ethics committee as part of the study protocol. The verbal consent process included a clear explanation of the study and participants' rights, and consent was audio-recorded with participants’ permission. All interviews were conducted following COVID-19 safety guidelines and ethical standards for research involving human participants.

## Data Availability

The transcripts from the interviews contain sensitive information that could reveal the identities, views, and positions of participants, and sharing them would breach confidentiality agreements. Requests can be directed to the lead author at rajeevbr@iphindia.org; however, complete data access cannot be guaranteed. Requests will only be considered if substantial redactions can be made to safeguard participant anonymity.
