# Supplementary material for: Strengthening Governance for Universalising Primary Oral Health Care: Perspectives from Karnataka, India
Source: Wellcome Open Res. 2025 Oct 10;10:305. Originally published 2025 Jun 2. [Version 2] doi: 10.12688/wellcomeopenres.24152.2 (PMC12595295; doi:10.12688/wellcomeopenres.24152.2)
Supplement: Peer review history

# Reviewer report: Reviewer response for version 2

**DOI:** 10.21956/wellcomeopenres.27672.r137575

**Published:** 2025-11-08

**Authors:** Melisa Öçbe

**Affiliations:** 1 Kocaeli Health and Technology University, Kocaeli,, Turkey

This study assessed the governance structure and systemic challenges of oral healthcare delivery in Karnataka, India, with Siddiqi’s Health Governance Assessment Framework. This study includes qualitative interviews with 20 key stakeholders. The aim is to explore barriers and enablers affecting oral health governance, policy implementation, and alignment with Universal Health Coverage (UHC) principles.

 The topic is both timely and underexplored. Governance analysis of oral health systems is rare, especially in low- and middle-income countries. The focus on Karnataka provides valuable findings for national policy adaptation.

 Although snowball sampling sounds a little bir restricted, I think the final participant count & diversity is acceptable. One thing maybe considered in future studies: including the patients? Lack of patient as participants may be stated as a limitation. 

 As a quantitative feature, there might be some parameters such as patient statistics including the current oral health status with indices, policy analysis etc. 

 The discussion of financial transparency focuses on budget delays and fund utilization, but not sufficiently on decision-making visibility, public access to information, or citizen oversight mechanisms—all key elements of governance.

 Findings are grounded in Karnataka’s health governance structure, limiting generalizability to other Indian states without adaptation to local institutional contexts. 

 Not mentioned in the text: oral cancer burden of India, especially in southern states. Oral potentially malignant lesions (due to betel nut, areca nut) and oral squamous cell carcinoma are seriously increased in India. This should be stated in Discussion, with addition of relevant references. The main point here is, initially may a survey reflects the awareness and knowledge level (there are many in the literature) and in other stages, oral cancer screening should be applied to large populations (again, not rare in India). After detecting the burden, diagnosis and treatment strategies should be discussed.

Is the work clearly and accurately presented and does it cite the current literature?

Yes

If applicable, is the statistical analysis and its interpretation appropriate?

Not applicable

Are all the source data underlying the results available to ensure full reproducibility?

Partly

Is the study design appropriate and is the work technically sound?

Yes

Are the conclusions drawn adequately supported by the results?

Partly

Are sufficient details of methods and analysis provided to allow replication by others?

Yes

Reviewer Expertise:

Oral and Maxillofacial Radiology, Oral Medicine, Oral Oncology

I confirm that I have read this submission and believe that I have an appropriate level of expertise to confirm that it is of an acceptable scientific standard.

# Reviewer report: Reviewer response for version 1

**DOI:** 10.21956/wellcomeopenres.26643.r131688

**Published:** 2025-09-23

**Authors:** Venkitachalam Ramanarayanan

**Affiliations:** 1 Department of Public Health Dentistry, Amrita School of Dentistry, Kochi, Kerala, India

Congratulations on a well-written paper. Governance is one of the major building blocks of health systems. Governance is very unique as it also influences several other building blocks like finance, human resources, service delivery etc. This paper captures this information in Karnataka quite comprehensively.

 Few observations on the paper

 - There are many programs on oral health which have been mentioned. Is there any information on the current status of the program. For eg, Danta bhagya, is it still being implemented? any barriers in implementation of these program (national policies) etc

 - Among the different types of stakeholders, most of them have only been a single respondent. I feel stating that data collection was continued till data saturation might not seem appropriate. The study could have included more of private practitioner or academicians to capture differing views. 

 - The study included 5 administrators. Could you provide more details on the designation of the administrators? Was there an attempt to contact high ranking officials like Minister for Health or secretary of health?

 -  Has there been any study regionally or globally capturing the governance or similar aspects of oral health care delivery ? If so, it could be mentioned in the discussion for comparison.

Is the work clearly and accurately presented and does it cite the current literature?

Yes

If applicable, is the statistical analysis and its interpretation appropriate?

Not applicable

Are all the source data underlying the results available to ensure full reproducibility?

Yes

Is the study design appropriate and is the work technically sound?

Yes

Are the conclusions drawn adequately supported by the results?

Yes

Are sufficient details of methods and analysis provided to allow replication by others?

Yes

Reviewer Expertise:

Dental Public Health, Health systems research

I confirm that I have read this submission and believe that I have an appropriate level of expertise to confirm that it is of an acceptable scientific standard.

# Reviewer report: Reviewer response for version 1

**DOI:** 10.21956/wellcomeopenres.26643.r125064

**Published:** 2025-08-21

**Authors:** Beverley Essue, Diego Proano Falconi

**Affiliations:** 1 Dalla Lana School of Public Health, West Toronto, Canada; 2 University of Toronto Institute of Health Policy Management and Evaluation (Ringgold ID: 206712), Toronto, Ontario, Canada; 3 Faculty of Dentistry, University of Toronto (Ringgold ID: 7938), Toronto, Ontario, Canada

Thank you for this opportunity to review this paper. we have outlined details below that we believe are needed to strengthen the paper and finalize it for publication.

 Major Comments

 Introduction

- The connection between the global neglect of oral health and the specific case of India is not sufficiently clear in the first few sentences. I suggest the authors revise the opening to more explicitly situate India within a broader pattern of global under-prioritization, or to clearly articulate why India is being highlighted uniquely.
- The final sentence of the first paragraph refers to “Universal Oral Health Coverage.” This phrasing could cause confusion, as WHO and UN documents generally refer to “Universal Health Coverage for Oral Health.” If the authors are referring to the same concept, the terminology should be harmonized to avoid ambiguity. If referring to a distinct concept, this should be explained explicitly.
- The claim that Karnataka is recognized for a relatively robust health system should be supported by a citation or source (e.g., national rankings, performance metrics). This will strengthen the assertion and provide credibility.
- The authors should elaborate briefly on what constitutes Karnataka’s demographic and geographic diversity—whether referring to urban-rural divides, socio-economic gradients, or population heterogeneity—to clarify how this complexity challenges equitable health service delivery.
- The assertion that Karnataka requires “targeted interventions” raises a conceptual issue when considered alongside the universalist principles of UHC. The authors should clarify whether targeted interventions are intended to supplement universal strategies or address specific equity gaps, and how these align with the broader goal of UHC.
- The statement about a rising burden of oral health conditions should be substantiated with a reference—either from epidemiological studies, program data, or national surveillance reports. (e.g., fluorosis, oral cancer prevalence) would also help contextualize this claim.

Methods

- The snowball sampling approach could benefit from further elaboration. Specifically, the authors should explain how initial participants were identified, what criteria were used, and how they facilitated recruitment of subsequent interviewees or how diverse or representative the final sample is in terms of geography, gender, roles, or institutional affiliations. Without this, the potential for sampling bias remains high and unaddressed. Were efforts made to ensure representation from underserved or remote districts, or from private providers who may have different perspectives? This will increase transparency and methodological rigor.
- While the study employs an exploratory qualitative design, the authors should explicitly justify why this design was chosen over other potential qualitative or mixed methods approaches. For example, was the exploratory design selected due to the lack of existing empirical data on governance in oral health in India? A clearer rationale would strengthen the alignment between the research problem and method.
- More detail is needed on how participants were approached and informed about the study, especially since power differentials likely exist between participants (e.g., junior vs. senior administrators, or private vs. public sector professionals). Clarifying how voluntariness, confidentiality, and informed consent were assured would enhance the transparency of ethical practices.
- The claim that interviews were conducted “until saturation” is not substantiated. How did the researchers define and determine saturation? Was a saturation grid or iterative coding log used? A brief explanation of how saturation was operationalized would improve methodological rigor.
- The authors mention that the interview guide was developed using Siddiqi’s and Batchelor’s frameworks but do not report whether the guide was pilot tested or refined before deployment. Did they conduct cognitive interviews or trial runs? Given the diversity of participants, ensuring that the language was appropriate and understandable across roles is important.
- There is no discussion of the researchers’ positionality and how their backgrounds, affiliations, or assumptions may have influenced the interviews or data analysis. This is particularly important in governance research where power dynamics and institutional affiliations can shape responses.
- While the authors describe using a framework method and Excel for analysis, additional clarity is needed on how themes were developed and validated. For instance:
  - How were discrepancies between the two researchers resolved?
  - Were codes double-checked independently?
  - Was any qualitative software used to enhance coding consistency?
- Although policy and program documents were mentioned as part of the situational analysis, the paper does not explain how these documents were systematically reviewed or triangulated with interview data. Which documents were reviewed, how were they selected, and what analytical method (if any) was used?
- The limitations section mentions the exclusion of the private sector, but this is only partially accurate. One private dental practitioner is included. Instead, a more substantive methodological limitation is the overrepresentation of government actors, potentially limiting insight into governance tensions, implementation bottlenecks, or coordination failures with private sector stakeholders. This imbalance should be acknowledged more directly.

Results

- In the “Governance Factors” section, the narrative sometimes blends direct participant perspectives with the authors' interpretation. For clarity, I recommend clearly delineating quotes and descriptive findings from analytical or interpretative content. Any thematic analysis or interpretation should be relocated to the Discussion section.

- The manuscript does not specify whether any form of validation (e.g., member checking, triangulation) was used to strengthen credibility. If validation was conducted, it should be stated. If not, a brief rationale for this omission would be appropriate.
- The content under the “Transparency” domain appears to focus more on financial planning and budget execution rather than transparency in the sense of open access to information, accountability, or decision-making visibility. The authors should either clarify how the content aligns with the governance principle of transparency or consider reframing this section.

Minor Comments

- The introduction currently opens with definitive statements about the health and socio-economic implications of oral disease. These claims would be more precise if hedged slightly. Suggested edits:
  - “Oral diseases… may have significant implications for overall health.”
  - “Dental diseases may impose a substantial socio-economic burden, particularly under certain conditions.”
- The first instance of “UHC” should be preceded by the full term “Universal Health Coverage.”
- In the discussion, the statement that dental procedures range from “15–45 minutes” would benefit from a citation or clarification that this is an approximate generalization based on expert opinion.
- Reference 6: The second word in the title of Reference 6 is capitalized erroneously (“HEalth”). This should be corrected.

Is the work clearly and accurately presented and does it cite the current literature?

Partly

If applicable, is the statistical analysis and its interpretation appropriate?

Partly

Are all the source data underlying the results available to ensure full reproducibility?

Partly

Is the study design appropriate and is the work technically sound?

Partly

Are the conclusions drawn adequately supported by the results?

Partly

Are sufficient details of methods and analysis provided to allow replication by others?

Partly

Reviewer Expertise:

Global health systems research; health economics; health policy; equity' access; health financing

We confirm that we have read this submission and believe that we have an appropriate level of expertise to confirm that it is of an acceptable scientific standard, however we have significant reservations, as outlined above.
